# Editorial: Curcuminoids: their pleiotropism against hallmarks of cancers

**DOI:** 10.3389/fphar.2023.1266793

**Published:** 2023-08-18

**Authors:** Daniel L. Pouliquen, Koraljka Gall Trošelj, Ruby John Anto, Rakesh Naidu

**Affiliations:** ^1^ Inserm, CNRS, Nantes Université, CRCI2NA, Université d’Angers, Angers, France; ^2^ Laboratory for Epigenomics, Division of Molecular Medicine, Ruđer Bošković Institute, Zagreb, Croatia; ^3^ Division of Cancer Research, Rajiv Gandhi Centre for Biotechnology, Thiruvananthapuram, India; ^4^ Jeffrey Cheah School of Medicine and Health Sciences, Monash University Malaysia, Jalan Lagoon Selatan, Bandar Sunway, Malaysia

**Keywords:** curcumin, curcumin analogs, molecular targets, signaling pathways, nano-formulations, molecular mechanism, clinical trials, combined therapy

Curcuminoids (natural and synthetic diarylheptanoids) represent a growing topic in cancer research, with 5,562 references reported in the Pubmed database since 1983. During the past two decades, the number of annually published articles has been continuously raising, reaching more than 450 papers per year between 2018 and 2021, and including more than 80 annual reviews. A wealth of studies has documented pleiotropic effects of curcumin against hallmarks of cancers both *in vitro* and *in vivo*, and new analogs, formulations, and innovative nanomaterials offering improved bioactivity, in addition to its historical leading molecule, curcumin, are currently under development (Lu et al., 2023). Therefore, this Research Topic aimed at providing an updated overview of some aspects of this research, highlighting the complexity of their mechanisms of action, and addressing several important questions that are relevant for a potential future translation of experimental data into clinical studies.

For dealing with the previously existing gap between basic scientists and clinicians regarding the potential of curcumin for clinical applications, Shafei et al. reviewed its role, when administered alone or in combination with other chemotherapeutics in the treatment of colorectal cancer (CRC). They pointed out to the clinical advantage that curcumin would manifest if administered through currently developing formulations. Besides the expected reduction of side-effects associated with the use of standard chemotherapeutic that curcumin might provide in the treatment of metastatic CRC (Layos et al., 2022), and its effects in prevention (Weng and Goel, 2022), they reported that this evolution could lead to an almost 40-fold increase in the concentration of this molecule in the blood.

Curcuminoids are characterized by multiple and simultaneous effects on interconnected signaling pathways sustaining permanent proliferation. New molecular targets are continuously being discovered, and among them are many drivers of the epithelial-to-mesenchymal transition (EMT), which orchestrate various features of cancer invasiveness. Pouliquen et al. reviewed this growing topic, pointing out to the great diversity of signaling pathways involved in this process. The crucial role of non-coding RNAs, and numerous components of the tumor microenvironment was also emphasized. Finally, new insights provided by investigations on experimental models of peritoneal malignant mesothelioma designed in immunocompetent rats were thoroughly discussed.

MicroRNAs targeted by curcuminoids are evolving as an important topic, due to their involvement in the occurrence of chemoresistance. Curcuminoids alone are well-known for their anticancer effects, and their synergism with the conventional anti-cancer drugs is also well documented. Experimental treatment of ovarian cancer cells with combination of curcumin and paclitaxel resulted in a synergistic effect. The synergy was also observed *in vivo*, resulting in a strong suppression of tumor xenografts. In this experimental model, Liu et al. identified BRCA1-targeting miR-9-5p as a new curcumin molecular target. In agreement with this discovery is the recently published paper by Zhu et al., who revealed that hypoxia-induced miR-9 expression is indeed involved in the progression of this cancer type via the PI3K/AKT/mTOR/GSK3β pathway (Zhu et al., 2023).

Since 2008, the impressive development of network pharmacology applied to anticancer drugs has led to questioning the dominant paradigm in drug discovery that was represented by the concept of “designing maximally selective ligands to act on individual drug targets” (Hopkins, 2008). The polypharmacology approach progressively led to identification of highly complex deregulated networks in cancer cells exposed to natural drugs (Poornima et al., 2016). Bioinformatics also started to be applied for investigating the pleiotropic effects of curcumin (Wang et al., 2018). To improve our knowledge in this field, two important breakthroughs were presented in this Research Topic. First, with respect to the report discussed earlier—regarding the effects of curcumin on CRC, He et al. have thoroughly analyzed curcumin-related effects through exploration of numerous publicly available datasets and molecular docking. They were able to profile 73 potential targets, and 34 signaling pathways, of which the metabolic pathway was shown to be severely affected. Secondly, with respect to previous findings related to the effects of curcumin on breast cancer cells, Deng et al., using a combination method of network pharmacology, molecular docking and *in vitro* experiments, have investigated the diversity of molecular targets of curcumin in triple negative breast cancer (TNBC). They identified forty potential TNBC targets of curcumin, demonstrating the pharmacological effects of both curcumin and its nano-formulation through the downregulated expression of 10 main targets.

Several other major constituents of the natural main source of curcumin, the dried root of Curcuma longa, have been the subject of many investigations during the past two decades. Among them, curcuphenol, which is a bisabolane-type sesquiterpenoid, originally showed anti-inflammatory and antimicrobial properties that are comparable to those identified for curcumin, prior to the first demonstrations of its anticancer properties (Kuttan et al., 1987). Like curcumin, curcuphenol is a specialized metabolite, which is not directly involved in the growth or development of the organisms producing them. However, convergent efforts toward an understanding of the biosynthesis of these molecules in all kingdoms of life are under way, to mine their chemical diversity for health purposes (Nair and Jez, 2020). Intriguingly, the interest for curcuphenol in cancer research has emerged initially from pioneering studies conducted on marine sponges three decades ago. In this Research Topic, Nohara et al. have revealed that this molecule, identified via the screening of 480 marine invertebrate extracts, contributes to reversion of the downregulation of the antigen processing and presentation machinery, a phenomenon which is observed in the transition of primary tumors to their metastatic forms. Additionally, Ellis et al. have demonstrated HDAC (histone deacetylase)-modifying activities in curcuphenol analogues that able to induce the expression of antigen presentation machinery (APM) which in fact reverses the immune-escape phenotype in metastatic tumour cells. This suggests that these analogues may indicate anti-metastatic activity by modulating the immune responses through epigenetic means.
